# Self-perceived weather sensitivity and joint pain in older people with osteoarthritis in six European countries: results from the European Project on OSteoArthritis (EPOSA)

**DOI:** 10.1186/1471-2474-15-66

**Published:** 2014-03-05

**Authors:** Erik J Timmermans, Suzan van der Pas, Laura A Schaap, Mercedes Sánchez-Martínez, Sabina Zambon, Richard Peter, Nancy L Pedersen, Elaine M Dennison, Michael Denkinger, Maria Victoria Castell, Paola Siviero, Florian Herbolsheimer, Mark H Edwards, Ángel Otero, Dorly JH Deeg

**Affiliations:** 1 Department of Epidemiology and Biostatistics, EMGO+ Institute for Health and Care Research, VU University Medical Center, Van der Boechorststraat 7, 1081 BT, Amsterdam, the Netherlands; 2 Department of Preventive Medicine and Public Health, Unit of Primary Care and Family Medicine, Faculty of Medicine, Universidad Autonoma de Madrid, Arzobispo Morcillo 4, 28029 Madrid, Spain; 3 Department of Medicine, University of Padova, Via 8 Febbraio 2, 35122 Padova, Italy; 4 National Research Council, Aging Branch, Institute of Neuroscience, Via Giustiniani 2, 35128 Padova, Italy; 5 Institute of the History, Philosophy and Ethics of Medicine, University of Ulm, Frauensteige 6, 89075 Ulm, Germany; 6 Department of Medical Epidemiology and Biostatistics, Karolinska Institutet, P.O.Box 281, Nobels väg 12A, SE-171 77 Stockholm, Sweden; 7 MRC Lifecourse Epidemiology Unit, University of Southampton, Southampton General Hospital, Tremona Road, Southampton SO16 6YD, United Kingdom; 8 Bethesda Geriatric Clinic, University of Ulm, Zollernring 26, 89073 Ulm, Germany

**Keywords:** Europe, Joint pain, Older people, Osteoarthritis, Weather sensitivity

## Abstract

**Background:**

People with osteoarthritis (OA) frequently report that their joint pain is influenced by weather conditions. This study aimed to examine whether there are differences in perceived joint pain between older people with OA who reported to be weather-sensitive versus those who did not in six European countries with different climates and to identify characteristics of older persons with OA that are most predictive of perceived weather sensitivity.

**Methods:**

Baseline data from the European Project on OSteoArthritis (EPOSA) were used. ACR classification criteria were used to determine OA. Participants with OA were asked about their perception of weather as influencing their pain. Using a two-week follow-up pain calendar, average self-reported joint pain was assessed (range: 0 (no pain)-10 (greatest pain intensity)). Linear regression analyses, logistic regression analyses and an independent t-test were used. Analyses were adjusted for several confounders.

**Results:**

The majority of participants with OA (67.2%) perceived the weather as affecting their pain. Weather-sensitive participants reported more pain than non-weather-sensitive participants (M = 4.1, SD = 2.4 versus M = 3.1, SD = 2.4; p < 0.001). After adjusting for several confounding factors, the association between self-perceived weather sensitivity and joint pain remained present (B = 0.37, p = 0.03). Logistic regression analyses revealed that women and more anxious people were more likely to report weather sensitivity. Older people with OA from Southern Europe were more likely to indicate themselves as weather-sensitive persons than those from Northern Europe.

**Conclusions:**

Weather (in)stability may have a greater impact on joint structures and pain perception in people from Southern Europe. The results emphasize the importance of considering weather sensitivity in daily life of older people with OA and may help to identify weather-sensitive older people with OA.

## Background

Osteoarthritis (OA) is a degenerative joint disease, which is mainly characterized by damage and loss of articular cartilage and changes in adjacent bone, including osteophytes and subchondral bone sclerosis [1,2]. OA is the most common cause of chronic pain in older persons and the leading cause of disability [3]. People with OA frequently report that the severity of their pain is influenced by weather conditions [4]. The impairment of well-being and/or incidence of symptoms or exacerbations of diseases related to weather is termed weather sensitivity [5]. Research on the effect of self-perceived weather sensitivity on joint pain in people with OA is scarce. Knowledge gained on the perceived influence of weather on joint pain in older people with OA could be applied in the development of coping strategies for dealing with joint pain and climatologic conditions in this disease group. The present study aims to examine whether there are differences in joint pain between older people with OA who reported to be weather-sensitive and those who did not in six European countries with different climates.

Research on the perceived influence of weather on pain is mainly conducted in chronic pain patients and diagnostic differences in subgroups of patients are rarely examined [6,7]. Jamison et al. [6] investigated the differences in the perceived influence of weather on pain among 558 chronic pain patients, living in four different climates in the United States of America (USA). The most frequent complaints in this group were lower back pain and arthritis. Cold and damp weather conditions were perceived to influence pain most. The weather-sensitive and non-weather-sensitive chronic pain patients did not differ in self-reported pain intensity. Notably, chronic pain patients who had been told that they had arthritis tended to report greater weather sensitivity to weather changes. However, the diagnoses were based solely on self-report, without objective medical confirmation.

In an Australasian study, Ng et al. [8] found that the majority of OA-patients reported weather sensitivity. Various physiological and psychological explanations have been offered for the greater sensitivity to weather in OA-patients [6,8-10]. It has been suggested that because tendons, muscles, bones and scar tissues are of varied densities, differential expansions and contractions due to atmospheric changes results in pain at sites of microtrauma [6,8]. In addition, alterations in temperature may increase stiffness in the joints and may trigger subtle movements that can heighten a nociceptive response [6,9]. It has also been suggested that weather affects mood, resulting in an alteration of pain perception [6,8-10]. Negative mood is associated with high levels of pain in people with OA [11,12]. Rainy weather conditions may adversely affect mood and thus may indirectly affect pain perception.

The specific objectives of the present study are: (1) to examine whether there are differences in perceived joint pain between weather-sensitive and non-weather-sensitive people with OA in six European countries with different climates; and (2) to identify characteristics of older persons with OA that are most predictive of perceived weather sensitivity.

## Methods

### Design and study sample

Baseline data from the European Project on OSteoArthritis (EPOSA) were used. The EPOSA study focuses on the personal and societal burden of OA and its determinants in older persons. A detailed description of the study design and data collection of the EPOSA study is described elsewhere [13]. In summary, random samples were taken from existing population-based cohorts in five European countries (Germany, the Netherlands, Spain, Sweden and the United Kingdom (UK)). In Italy, a new sample was drawn. A total of 2942 respondents (response rate, ranging from 64.6% to 82.2%, averaging 72.8%) were included. The age-range was between 65-85 years in most countries except for the UK, which had an age-range of 71-80 years. All participants were interviewed by a trained researcher at home or in a clinical center, using a standardized questionnaire and a clinical exam. The interview lasted about one and a half hours. All participants completed an informed consent. For all six countries, the study design and procedures were approved by the Medical Ethics committee of the respective centers (Germany: Ethical Committee of Ulm University; the Netherlands: Medical Ethical Committee of the VU University Medical Center; Spain: Ethic Committee for Clinical Research of University Hospital La Paz of Madrid; Sweden: Ethics Board of Karolinska Institutet; UK: The Hertfordshire Research Ethics Committee; Italy: Comitatio etico ULSS7).

In the EPOSA study, clinical classification criteria, developed by the American College of Rheumatology (ACR) [14], were used to determine clinical OA. The ACR criteria for any clinical knee, hip or hand OA was satisfied in 889 participants (31.7%). Of these participants, 727 persons completed all 14 days of the pain calendar. Data on self-perceived weather sensitivity was available for 712 subjects. These participants were included in the final study sample of the current study. The excluded participants with clinical OA (n = 177) were older, lower educated and more depressed than the included subjects. In addition, they had a lower sense of mastery and used less (additional) pain medication than the included participants. The two groups did not differ in sex, partner status, anxiety, body mass index (BMI), number of chronic diseases and outdoor physical activity.

### Measures

#### Dependent variable

##### Self-reported joint pain

Joint pain was assessed prospectively with a two-week pain calendar. After the baseline-interview, participants were asked to complete this pain calendar. Per day respondents indicated how much joint pain they experienced on a 11-point rating scale from 0 to 10 with 0 representing no pain and 10 representing the greatest pain intensity. For each respondent, the average self-reported joint pain in the pain calendar period was calculated as the sum of all noted pain intensity levels divided by 14.

#### Independent variable

##### Self-perceived weather sensitivity

To assess self-perceived weather sensitivity, participants were asked which specific weather condition(s) affects their joint pain. There were four response categories: my joint pain is affected by (1) damp/rainy weather, (2) cold weather, (3) hot weather, and (4) my joint pain is not affected by one of these weather conditions. Participants were allowed to indicate more than one answer. Participants were considered as weather-sensitive persons when, in their opinion, damp/rainy, cold and/or hot weather affected their joint pain. Subjects who noted that their joint pain is not affected by one of these weather conditions were considered as non-weather-sensitive persons.

#### Potential confounders

##### Socio-demographic variables

Prior studies revealed that socio-demographic factors are associated with pain intensity in people with OA [12]. Socio-demographic information was obtained on participants’ age, sex, partner status and education level. Partner status referred to whether participants have a partner at the moment (yes/no). Education was measured by the highest level of education completed (elementary school not completed, elementary school completed, vocational education/general secondary education, and college or university education) and dichotomised into “better educated than secondary education” (yes/no).

### Pain medication use

Pain medication use (yes/no) referred to the use of analgesics (ATC N02 subgroup) and/or anti-inflammatory products (ATC M01 subgroup). In addition, participants were asked whether they used additional pain medication on the day of pain report because of joint pain. For each participant, the total number of days on which they used additional pain medication was calculated.

### Emotional distress: anxiety and depression

Emotional distress, such as anxiety and depression, is associated with more pain in people with OA [11,12]. Anxiety and depressive symptoms were examined by the Hospital Anxiety Depression Scales (HADS) [15]. HADS is a self-report questionnaire comprising 14 four-point Likert scaled items, 7 for anxiety (HADS-A) and 7 for depression (HADS-D). Both scales have a range from 0 to 21. A higher score on the HADS-A and HADS-D indicates greater anxiety and depression respectively.

### Mastery

Mastery is the extent to which individuals consider themselves to be in control of events and ongoing situations [16]. Mastery is considered as a psychological resource when coping with stressful life events. A high sense of mastery reduces psychological distress and therefore it may affect pain perception in people with OA.

Mastery was measured by means of an abbreviated 6-item version of the Pearlin Mastery Scale [16]. The questionnaire consists of six statements such as “I can do almost everything, if I want to”. Response categories range from 1 = strongly disagree to 5 = strongly agree. The summed items range from 6 to 30, but for ease of interpretation 6 is subtracted, so the final scale ranges from 0 to 24, with higher scores indicating more mastery.

### Outdoor physical activity

It has been shown that physical activity is beneficial for reducing pain in people with OA [17]. Physical activity was measured using the LASA Physical Activity Questionnaire (LAPAQ), an instrument validated against diaries and pedometer measurements in older persons [18]. Frequency and duration of activities over the past two weeks were asked for walking, cycling, gardening, light and heavy household work and a maximum of two sports. In order to calculate the daily outdoor physical activity, the frequency and duration of walking, cycling and gardening were multiplied and divided by 14 days. A total outdoor activity score was calculated in minutes per day.

### Body mass index

Body mass index (BMI) affects pain in OA-patients. Pain increases with patients’ weight [19]. BMI was calculated as weight in kilograms divided by height in squared meters. Weight was measured to the nearest 0.1 kg using a calibrated scale. Height was measured to the nearest 0.001 m using a stadiometer.

### Number of chronic diseases

It has been shown that number of comorbid conditions, including chronic diseases, influences pain in OA-patients [12]. Number of chronic conditions was measured through self-reported presence of the following chronic diseases or symptoms that lasted for at least three months or diseases for which the participant had been treated or followed by a physician: chronic non-specific lung disease, cardiovascular diseases, peripheral artery diseases, stroke, diabetes, cancer, and osteoporosis. If participants answered “yes” then they were asked to specify which diseases or type. Chronic conditions were evaluated as the number of diseases and multimorbidity was defined as the occurrence of 2 or more coexisting conditions.

### Local climate

Local climate of the residences of the participants in the six population-based cohort studies were classified by the Köppen-Geiger climate classification system. The Köppen-Geiger climate classification system is applied in various disciplines and is the most frequently used climate classification system in the world [20]. Based on criteria about vegetation, annual and monthly precipitation and temperature, this classification system distinguishes thirty possible climate types [21]. In the current study, three different climate types were classified. The residence locations of the participants in Germany, Italy, the Netherlands and the UK are characterized by a temperate warm climate without dry seasons and a warm summer (relatively warm and wet climate). The residence location in Spain is characterized by a temperate warm climate with a dry and hot summer (relatively warm and dry climate). The Swedish residence locations represent a cold climate without dry seasons and a warm summer (relatively cold and wet climate).

### Seasonal weather patterns

Seasonal weather patterns affect pain perception in weather-sensitive people. Additionally, weather patterns may influence mood in certain individuals and thereby indirectly affect pain perception [9,10]. The season (spring, summer, autumn or winter) in which the pain calendar is completed by the participant may have an effect on pain perception in older people with clinical OA. Information was obtained concerning the astronomical season in which participants completed their pain calendar.

### Statistical analyses

Differences in characteristics between weather-sensitive and non-weather-sensitive participants were examined with independent sample t-tests for continuous data and chi-square tests for categorical data. Differences between weather-sensitive and non-weather-sensitive persons were tested with a Mann-Whitney U test for skewed continuous variables. Descriptive analyses were used to examine the percentages of weather-sensitive persons who reported to be sensitive to a particular weather condition or a combination of specific weather conditions.

To examine differences in self-reported joint pain between weather-sensitive and non-weather-sensitive people with clinical OA, an independent sample t-test was performed. Self-perceived weather sensitivity and self-reported joint pain were used as independent and dependent variable respectively. Linear regression analyses were performed to correct for socio-demographic characteristics (sex, age, partner status, education and country) and other potential confounders (anxiety, depression, mastery, outdoor physical activity, medication use, BMI, number of chronic diseases, seasonal weather patterns and local climate).

Logistic regression analyses were performed to determine those variables that best predicted self-perceived weather sensitivity. First, each variable was examined for significantly predicting self-perceived weather sensitivity. Subsequently, all variables with a p-value below 0.20 were included in a multivariable model. Level of significance was α = 5.0%. Statistical analyses were performed in IBM SPSS Statistics (version 20.0).

## Results

The mean age of all 712 participants with OA was 73.5 (SD = 5.5) years. Of all participants, 484 (72.0%) were female and 469 (67.2%) participants reported that weather affects their joint pain.

The characteristics of weather-sensitive and non-weather-sensitive participants are presented in Table 1. The weather-sensitive participants were more often female and lower educated. They had a lower sense of mastery and were more anxious and depressed compared to the non-weather-sensitive participants. The weather-sensitive participants used additional pain medication on more days than the non-weather-sensitive participants. Weather-sensitive and non-weather-sensitive subjects did not differ in age, partner status, BMI, number of chronic diseases and outdoor physical activity.

**Table 1:** **Characteristics of the study sample (n = 712) stratified for weather sensitivity**^**1**^

	**Weather-sensitive participants**	**Non-weather-sensitive participants**	**p-value**
	**(n = 469)**	**(n = 243)**	
**Socio-demographic characteristics**			
Age in years (Mean (SD) (range))	73.4 (5.6) (65–85)	73.7 (5.3) (65–85)	0.46
Sex (female) (n (%))	339 (75.9)	145 (64.0)	<0.01
Education (≥ secondary education) (n (%))	199 (42.9)	150 (61.1)	<0.001
Partner status (yes) (n (%))	305 (64.2)	159 (63.9)	0.91
**Psychological characteristics and physical activity**			
Total HADS–A score (0–21) (mean (SD))	6.5 (4.3)	4.9 (3.7)	<0.001
Total HADS–D score (0–21) (mean (SD))	4.8 (3.5)	4.0 (3.4)	<0.01
6–item Pearlin Mastery score (0–24) (mean (SD))	15.5 (5.1)	17.0 (4.7)	<0.001
Outdoor physical activity in minutes per day (median (IQR))	40.2 (18.6–84.2)	42.9 (21.4–74.6)	0.72
**Health characteristics and body composition**			
Pain medication use (yes) (n (%))	210 (44.8)	95 (39.8)	0.20
Number of days with additional pain medication (0–14) (mean (SD))	5.4 (5.7)	3.8 (5.2)	<0.001
Length in meters (mean (SD))	1.62 (0.09)	1.63 (0.10)	0.02
Weight in kilograms (mean (SD))	74.5 (14.4)	76.7 (14.7)	0.06
BMI in kg/m^2^ (mean (SD))	28.5 (5.0)	28.8 (5.2)	0.55
Chronic diseases (≥2) (n (%))	155 (32.6)	75 (32.1)	0.92
**Local climate**			
Local climate: Cold and wet (n (%))	83 (56.9)	66 (43.1)	–
Local climate: Warm and wet (n (%))	272 (67.7)	145 (32.3)	–
Local climate: Warm and dry (n (%))	114 (76.6)	32 (23.4)	–
**Joint pain perception**			
Self–reported joint pain (0–10) (mean (SD))	4.1 (2.4)	3.1 (2.4)	<0.001
Country: Sweden	2.7 (1.9)	2.0 (2.0)	0.04
Country: Germany	3.6 (2.1)	3.1 (1.4)	0.28
Country: Italy	4.3 (2.3)	4.0 (2.4)	0.57
Country: The Netherlands	4.0 (1.9)	3.0 (2.1)	0.01
Country: Spain	5.4 (2.5)	5.1 (2.8)	0.48
Country: United Kingdom	4.1 (2.6)	2.9 (2.3)	0.04
Season: Spring	4.4 (2.3)	3.5 (2.5)	<0.01
Season: Summer	4.0 (2.3)	2.8 (2.4)	<0.01
Season: Autumn	3.4 (2.3)	2.5 (2.2)	0.03
Season: Winter	4.6 (2.7)	3.3 (2.6)	0.01

^1^Descriptive statistics are weighted, n is non–weighted. The sample size may vary for some variables, because of missing values.

### Self-reported joint pain

Participants who were weather-sensitive experienced significantly more joint pain than non-weather-sensitive subjects. Weather-sensitive and non-weather-sensitive participants reported an average self-reported joint pain of 4.1 (SD = 2.4) and 3.1 (SD = 2.4) respectively (see Table 1). In all six countries, the weather-sensitive participants reported higher joint pain intensities compared to the non-weather-sensitive subjects (see Table 1). After adjustment for socio-demographics and country only, the association between self-perceived weather sensitivity and joint pain remained present (B = 0.62, p < 0.01) (see Model 2 in Table 2). In a fully adjusted model including age, sex, education, partner status, country, (additional) medication use, anxiety, depression, mastery, outdoor physical activity, BMI, number of chronic diseases and seasonal weather patterns simultaneously, the association between weather sensitivity and joint pain was decreased (B = 0.37, p = 0.03) (see Model 3 in Table 2). If country was replaced by local climate in the fully adjusted model, the association between self-perceived weather sensitivity and joint pain was still significant (B = 0.47, p = 0.01).

**Table 2:** The association between self-perceived weather sensitivity and self-reported joint pain adjusted for potential confounders

**Models**	**B**	**Standard error**	**p-value**	**Explained variance (%)**
**Model 1**^**1**^				3.9
Self-perceived weather sensitivity	1.03	0.19	<0.001	
**Model 2**^**2**^				21.2
Self-perceived weather sensitivity	0.62	0.19	<0.01	
Age	0.04	0.02	0.01	
Sex: Male	Ref.	Ref.	Ref.	
Sex: Female	0.41	0.19	0.03	
Education: < Secondary education	Ref.	Ref.	Ref.	
Education: ≥ Secondary education	–0.38	0.20	0.05	
Partner status: No partner	Ref.	Ref.	Ref.	
Partner status: Partner	–0.24	0.18	0.19	
Country: Sweden	Ref.	Ref.	Ref.	
Country: Germany	1.01	0.35	<0.01	
Country: Italy	1.55	0.28	<0.001	
Country: The Netherlands	1.10	0.28	<0.001	
Country: Spain	2.52	0.28	<0.001	
Country: United Kingdom	1.09	0.32	<0.01	
**Model 3**^**3**^				44.1
Self–perceived weather sensitivity	0.37	0.17	0.03	
Age	0.01	0.02	0.76	
Sex: Male	Ref.	Ref.	Ref.	
Sex: Female	0.08	0.18	0.65	
Education: < Secondary education	Ref.	Ref.	Ref.	
Education: ≥ Secondary education	–0.17	0.18	0.36	
Partner status: No partner	Ref.	Ref.	Ref.	
Partner status: Partner	0.09	0.17	0.59	
Country: Sweden	Ref.	Ref.	Ref.	
Country: Germany	0.79	0.34	0.02	
Country: Italy	1.14	0.31	<0.001	
Country: The Netherlands	0.51	0.33	0.12	
Country: Spain	1.17	0.34	<0.01	
Country: United Kingdom	–0.01	0.34	0.98	
Pain medication: No use	Ref.	Ref.	Ref.	
Pain medication: Use	0.29	0.17	0.09	
Number of days with additional pain medication	0.20	0.02	<0.001	
Anxiety	–0.01	0.03	0.74	
Depression	0.03	0.03	0.42	
Mastery	–0.06	0.02	0.01	
Outdoor physical activity	<–0.01	<0.01	0.50	
BMI	<0.01	0.02	0.94	
Number of chronic diseases	0.29	0.08	<0.001	
Season: Autumn	Ref.	Ref.	Ref.	
Season: Spring	–0.11	0.24	0.64	
Season: Summer	0.26	0.25	0.31	
Season: Winter	–0.52	0.29	0.08	

^1^The association between self–perceived weather sensitivity and self–reported joint pain unadjusted for potential confounders.

^2^The association between self–perceived weather sensitivity and self–reported joint pain adjusted for socio–demographics and country only.

^3^The association between self–perceived weather sensitivity and self–reported joint pain adjusted for socio–demographics, country and other potential confounders.

### Self-perceived weather sensitivity and local climate

Among the 469 weather-sensitive participants, 184 (39.2%) participants were sensitive to damp/rainy weather conditions, 145 (30.2%) participants reported to be only sensitive to cold weather and 23 (4.6%) participants were sensitive to hot weather. One hundred seventeen participants (26.0%) were sensitive to more than one weather condition. Ninety-eight subjects (22.0%) were sensitive to damp/rainy and cold weather. Seven (1.5%) participants were sensitive to rainy/damp and hot weather and eight (1.6%) participants reported that they were sensitive to cold as well as warm weather conditions. Only four (0.9%) participants were sensitive to all three weather conditions: damp/rainy, cold and hot weather.

**Figure 1:**
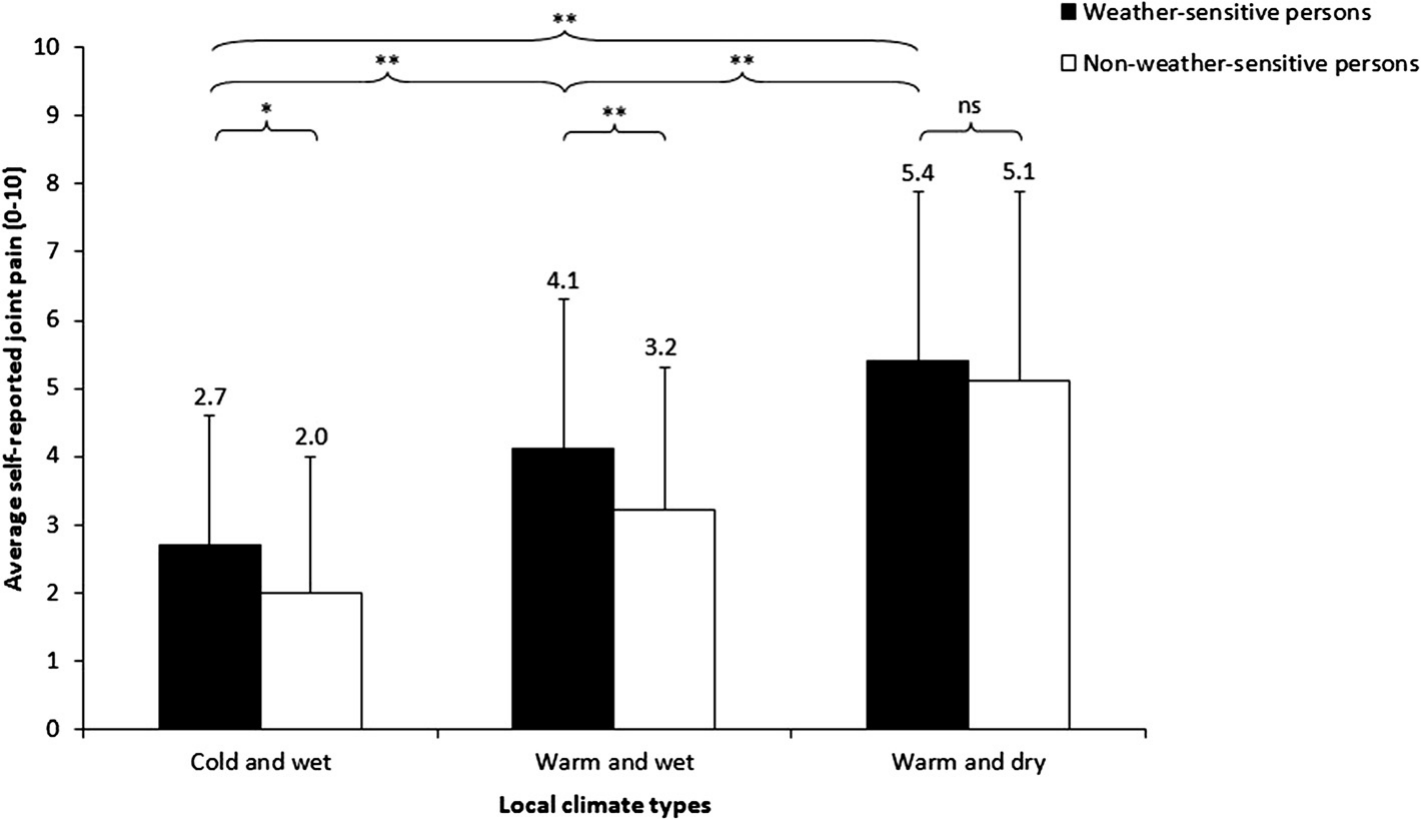
**Average self-reported joint pain of weather-sensitive and non-weather-sensitive persons in three climate types.** Descriptive statistics are weighted. Error bars represent one standard deviation of the mean. ^*^p < 0.05; ^**^p < 0.001; ns = not significant.

The percentage of weather-sensitive older people with OA was the highest in a warm and dry climate (76.6%) and the lowest in a cold and wet climate (56.9%) (see Table 1). In a cold and wet climate and a warm and wet climate, the weather-sensitive participants reported significantly higher joint pain intensity compared to the non-weather-sensitive participants (cold and wet climate: M = 2.7, SD = 1.9 versus M = 2.0, SD = 2.0; p = 0.04; warm and wet climate: M = 4.1, SD = 2.2 versus M = 3.2, SD = 2.1; p < 0.001; warm and dry climate: M = 5.4, SD = 2.5 versus M = 5.1, SD = 2.8; p = 0.48) (see Figure 1). The weather-sensitive participants in a warm and dry climate reported significantly higher joint pain intensity (M = 5.4, SD = 2.5) compared to those in a cold and wet climate (M = 2.7, SD = 1.9) and a warm and wet climate (M = 4.1, SD = 2.2) (p-values < 0.001) (see Figure 1). Weather-sensitive people with OA who were living in a warm and wet climate reported significantly a higher pain intensity level than those in a cold and wet climate (M = 4.1, SD = 2.2 versus M = 2.7, SD = 1.9; p < 0.001) (see Figure 1).

### Predictors of self-perceived weather sensitivity

As shown in Table 3, univariable models yielded six significant predictors of weather sensitivity in older persons with OA: sex, education, country, anxiety, depression and mastery (see Table 3). In a multivariable model including age and all significant predictors, sex, country and anxiety remained significant predictors of weather sensitivity in older people with OA (see Table 3). Women were more likely to report weather sensitivity than men. More anxious people with OA were more likely to be weather-sensitive than less anxious people with OA. Participants from Spain and Italy were more likely to indicate themselves as weather-sensitive persons compared to the subjects from Sweden.

**Table 3:** Predictors of self-perceived weather sensitivity in older persons with OA

**Predictors**	**Odds ratio**	**95%-confidence interval**	**p-value**
**Univariable models**			
Age	0.99	0.96-1.02	0.45
Sex: Male	Ref.	Ref.	Ref.
Sex: Female	1.77	1.26-2.49	<0.01
Education: < Secondary education	Ref.	Ref.	Ref.
Education: ≥ Secondary education	0.48	0.35-0.66	<0.001
Country: Sweden	Ref.	Ref.	Ref.
Country: Germany	1.35	0.72-2.54	0.35
Country: Italy	3.81	2.26-6.43	<0.001
Country: the Netherlands	1.23	0.75-2.02	0.41
Country: Spain	2.50	1.52-4.12	<0.001
Country: United Kingdom	0.65	0.37-1.12	0.12
Anxiety	1.11	1.06-1.16	<0.001
Depression	1.07	1.02-1.13	<0.01
Mastery	0.94	0.91-0.97	<0.001
**Multivariable model 1**			
Age	0.99	0.96-1.02	0.38
Sex: Male	Ref.	Ref.	Ref.
Sex: Female	1.94	1.35-2.79	<0.001
Education: < Secondary education	Ref.	Ref.	Ref.
Education: ≥ Secondary education	0.74	0.51-1.09	0.13
Country: Sweden	Ref.	Ref.	Ref.
Country: Germany	1.33	0.69-2.55	0.39
Country: Italy	3.70	2.09-6.55	<0.001
Country: the Netherlands	1.35	0.81-2.25	0.26
Country: Spain	2.31	1.33-3.99	<0.01
Country: United Kingdom	0.74	0.42-1.31	0.31
**Multivariable model 2**			
Age	0.99	0.96-1.03	0.61
Sex: Male	Ref.	Ref.	Ref.
Sex: Female	1.72	1.18-2.50	<0.01
Education: < Secondary education	Ref.	Ref.	Ref.
Education: ≥ Secondary education	0.74	0.50-1.10	0.14
Country: Sweden	Ref.	Ref.	Ref.
Country: Germany	1.43	0.71-2.87	0.32
Country: Italy	3.14	1.63-6.03	<0.01
Country: the Netherlands	1.36	0.76-2.46	0.30
Country: Spain	2.47	1.31-4.67	0.01
Country: United Kingdom	0.68	0.36-1.29	0.24
Anxiety	1.08	1.02-1.15	0.01
Depression	0.95	0.89-1.02	0.17
Mastery	1.00	0.95-1.05	0.95

## Discussion

This study aimed to examine whether there are differences in perceived joint pain between weather-sensitive and non-weather-sensitive people with OA in six European countries with different climates and to identify characteristics of older persons with OA that are most predictive of self-perceived weather sensitivity. The results confirmed that weather-sensitive older people with OA experience more joint pain than their non-weather-sensitive counterparts. Women and more anxious people were more likely to report weather-sensitivity. The results also revealed that older people with OA from Spain and Italy were more likely to report weather-sensitivity compared to those from Sweden.

Our study showed that weather-sensitive people with OA reported more pain than non-weather-sensitive persons with OA. After adjusting for several confounding factors, the association between self-perceived weather sensitivity and self-reported joint pain remained present. Previous research in chronic pain patients revealed conflicting results concerning differences in experienced pain between weather-sensitive and non-weather-sensitive patients [6,7]. In these studies, self-perceived weather sensitivity in subgroups of chronic pain patients was not examined. This study focused especially on older people with OA and confirmed that self-perceived weather sensitivity is related to pain perception in this specific group.

Our study further showed that approximately two thirds of the participants indicated themselves as weather-sensitive. Most of the weather-sensitive people with OA reported damp/rainy and/or cold weather as affecting their pain. Hot weather conditions were less frequently reported as influencing pain. Similar results have been found in previous studies with chronic pain patients and rheumatology patients [6,8]. Several explanations have been suggested to account for the effects of damp/rainy, cold and hot weather conditions on pain [6,8-10]. Changes in temperature and humidity may influence the expansion and contraction of different tissues in the affected joint, which may elicit a pain response [6,8]. In addition, low temperatures may increase the viscosity of synovial fluid, thereby making joints stiffer and perhaps more sensitive to the pain of mechanical stresses [6,9]. Another postulation is that weather affects mood, resulting in an alteration of pain perception [6,8-10]. This suggestion is not supported by our findings. The weather-sensitive people with OA were more anxious than those who were non-weather-sensitive. However, the association between self-perceived weather sensitivity and self-reported joint pain was still present after correcting for several confounders, including anxiety and depression. This suggests that emotional distress does not confound or mediate the association between self-perceived weather sensitivity and joint pain in older people with OA.

Although most weather-sensitive older people with OA reported to be sensitive to damp/rainy and/or cold weather, the common belief that joint pain in OA becomes worse by living in a cold and damp climate is not supported by our results. Our findings showed that weather-sensitive older people with OA in a cold and wet climate reported even lower pain intensity levels than those in a warm and wet or warm and dry climate. Jamison et al. [6] found that chronic pain patients in a colder climate did not report more pain than patients in warmer climates and suggested that the body establishes an equilibrium in relation to the local climate so that changes in weather trigger an increase in pain regardless of the prevailing meteorological conditions. Weather (in)stability might be an explanation for the differences in experienced joint pain between the three local climate types, however this was not assessed in this study.

Our findings showed that sex, country and anxiety are independent predictors of self-perceived weather sensitivity in older people with OA. It was found that women were more likely to report weather sensitivity than men. This seems to be in line with the findings of Von Mackensen et al. [5]. They found that women in the general population report a strong influence of weather on their health more often than men. Our results showed that more anxious people were more likely to indicate themselves as weather-sensitive persons. Possible explanations could be that poor mood might increase subjective complaints of pain or more anxious people with OA might tend to blame their symptoms on something they can understand but cannot control more than less anxious people with OA [9]. However, our findings showed that mastery is not an independent predictor of self-perceived weather sensitivity in older people with OA. The disease course of OA is often characterized by the alternation of stable periods of varying length, characterized by a low level or absence of symptoms with periods of flare-up or exacerbation [22]. The uncertainty about the recurrence of pain may lead to anxiety in people with OA and this might encourage the desire to have an explanation for the worsening of their pain. As a consequence, more anxious people with OA might be more likely to report weather as a pain-generating factor than less anxious people with OA.

Our findings also revealed that older people with OA from Spain and Italy were more likely to report weather sensitivity compared to older people with OA from Sweden. The climates in both Mediterranean countries are warmer compared to the climate in Sweden [21]. As a result, older people with OA in Italy and Spain may be more often outside compared to those in Sweden and the degree of exposure to the weather may vary between these people. As a consequence, they may be more aware of the effect of weather on their pain and are more likely to report weather sensitivity.

Another possible explanation might be differences in weather (in)stability between both Mediterranean countries and Sweden. Weather changes may have a greater impact on joint structures and pain perception in people from Southern Europe than in people from Northern Europe. As a result, people from Spain and Italy may be more aware of the effect of weather changes on their pain and are more likely to report weather sensitivity than people from Sweden.

There are several strengths in this study. To our best knowledge, the present study is the first large-scale study that examines self-perceived weather sensitivity and joint pain in older people with OA in Europe, correcting for a wide range of confounding factors. Prior studies were performed in the USA and Australasia and were mainly focused on self-perceived weather-sensitivity and pain in less specific groups [6-8]. The current study used a population-based approach and focused on one disease group. The assessment of clinical OA was standardized across countries using the ACR classification criteria. The current study increased insight into the characteristics profile of weather-sensitive people with OA in a general population of older persons across Europe. This may help to identify weather-sensitive older people with OA. Early treatment of weather-sensitive individuals with OA using cognitive and psychological interventions may reduce suffering and may help them to maintain a functionally effective lifestyle [23].

Some limitations of this study have to be acknowledged. Participants were considered as weather-sensitive persons, when they indicated that damp/rainy, cold, and/or hot weather affected their joint pain. If subjects noted that their joint pain was not affected by one of these weather conditions, they were considered as non-weather-sensitive persons. This classification method did not take into account whether participants’ joint pain could be affected by other weather conditions, such as changes in barometric pressure [6]. Furthermore, it is important to acknowledge some caveats with regard to the use of three local climate types. Two local climate types were only based on one country each. Spain represented a warm and dry climate and Sweden represented a cold and wet local climate. Only a warm and wet climate was represented by more than one country. Differences in experienced joint pain between the three climates may be due to other country-related factors. For example, differences in socio-cultural factors across countries may play a role [24,25].

Future research is needed to investigate actual versus perceived effects of weather on pain in weather-sensitive and non-weather-sensitive people with OA. In particular, longitudinal, prospective studies are needed to evaluate the relation of daily climatologic conditions to pain in older people with OA. The use of objective weather data may increase insight into the seasonal effects on joint pain in people with OA and the differences between countries.

## Conclusions

In conclusion, this study showed that the majority of older people with OA in the general population believe that weather affects their pain. Weather-sensitive older people with OA experience more joint pain than non-weather-sensitive older people with OA. Women and more anxious people are more likely to consider themselves as weather-sensitive. Older people with OA from Italy and Spain were more likely to report weather sensitivity than those from Sweden. Weather changes may have a greater impact on joint structures and pain perception in people from Southern Europe than in people from Northern Europe. The current results emphasize the importance of considering weather sensitivity in daily life of older people with OA and may help to identify weather-sensitive older people with OA.

## Abbreviations

ACR: American college of rheumatology; ATC: Anatomical therapeutic chemical; BMI: Body mass index; CI: Confidence interval; EPOSA: European project on osteoarthritis; HADS: Hospital anxiety depression scales; HADS-A: Hospital anxiety depression scales- anxiety subscale; HADS-D: Hospital anxiety depression scales- depression subscale; IBM SPSS Statistics: International business machines corporation statistical package for the social sciences statistics; IMCA – ActiFE: The Indicators for monitoring COPD and asthma – activity and function in the elderly in ulm study; LAPAQ: LASA physical activity questionnaire; LASA: Longitudinal aging study amsterdam; M: Mean; OA: Osteoarthritis; OR: Odds ratio; PNR: National research council project on aging; Ref: Reference category; SD: Standard deviation; UK: United Kingdom; USA: United States of America.

## Competing interests

The authors declare that they have no competing interests.

## Authors’ contributions

ET drafted the manuscript and performed the statistical analyses and interpreted the data. SvdP, LS and DD helped to draft the manuscript and contributed to the analysis and interpretation of data. MSM, SZ, RP, NP, ED, MD, MVC, PS, FH, ME and ÁO revised the manuscript critically for important intellectual content. All authors made substantial contributions to conception and design of the study and the acquisition of data. All authors also read and corrected draft versions of the manuscript and approved the final manuscript.

## Pre-publication history

The pre-publication history for this paper can be accessed here:

http://www.biomedcentral.com/1471-2474/15/66/prepub

## References

[B1] Arden N Nevitt MC Osteoarthritis: epidemiology Best Pract Res Clin Rheumatol 2006 20 3 25 10.1016/j.berh.2005.09.007 16483904

[B2] Woolf AD Pfleger B Burden on major musculoskeletal conditions Bull World Health Organ 2003 81 646 656 14710506 PMC2572542

[B3] Brooks PM Impact of osteoarthritis on individuals and society: how much disability? Social consequences and health economic implication Curr Opin Rheumatol 2002 14 573 577 10.1097/00002281-200209000-00017 12192258

[B4] Laborde JM Dando WA Powers MJ Influence of weather on osteoarthritics Soc Sci Med 1986 23 549 554 10.1016/0277-9536(86)90147-4 3764506

[B5] Von Mackensen S Hoeppe P Maarouf A Tourigny P Nowak D Prevalence of weather sensitivity in Germany and Canada Int J Biometerol 2005 49 156 166 10.1007/s00484-004-0226-2 15338386

[B6] Jamison RN Anderson KO Slater MA Weather changes and pain: perceived influence of local climate on pain complaint in chronic pain patients Pain 1995 61 309 315 10.1016/0304-3959(94)00215-Z 7659442

[B7] Shutty MS Cundiff G DeGood DE Pain complaint and the weather: weather sensitivity and symptom complaints in chronic pain patients Pain 1992 49 199 204 10.1016/0304-3959(92)90143-Y 1608646

[B8] Ng J Scott D Taneja A Gow P Gosai A Weather changes and pain in rheumatology patients APLAR J Rheumatol 2004 7 204 206 10.1111/j.1479-8077.2004.00099.x

[B9] Quick DC Joint pain and weather. A critical review of the literature Minn Med 1997 80 25 29 9090247

[B10] Sulman FG The impact of weather on human health Rev Environ Health 1984 4 83 119 6397793

[B11] Dekker J Boot B Van der Woude LHV Bijlsma JWJ Pain and disability in osteoarthritis: a review of biobehavioral mechanisms J Behav Med 1992 15 189 214 10.1007/BF00848325 1533877

[B12] Rosemann T Laux G Szecsenyi J Wensing M Grol R Pain and osteoarthritis in primary care: factors associated with pain perception in a sample of 1,021 patients Pain Med 2008 9 903 910 10.1111/j.1526-4637.2008.00498.x 18702636

[B13] Van der Pas S Castell MV Cooper C Denkinger M Dennison EM Edwards MH Herbolsheimer F Limongi F Lips P Maggi S Nåsell H Nikolaus T Otero A Pedersen NL Peter R Sanchez-Martinez M Schaap LA Zambon S Van Schoor NM Deeg DJH European project on osteoarthritis: design of a six-cohort study on the personal and societal burden of osteoarthritis in an older European population BMC Musculoskelet Disord 2013 14 138 10.1186/1471-2474-14-138 23597054 PMC3637520

[B14] Altman RD Classification of disease: osteoarthritis Semin Arthritis Rheum 1991 20 40 47 10.1016/0049-0172(91)90026-V 1866629

[B15] Zigmond A Snaith R The hospital anxiety and depression scale Acta Psychiatr Scand 1983 67 361 370 10.1111/j.1600-0447.1983.tb09716.x 6880820

[B16] Pearlin LI Schooler C The structure of coping J Health Soc Behav 1978 19 2 21 10.2307/2136319 649936

[B17] Veenhof C Huisman PA Barten JA Takken T Pisters MF Factors associated with physical activity in patients with osteoarthritis of the hip or knee: a systematic review Osteoarthritis Cartilage 2012 20 6 12 10.1016/j.joca.2011.10.006 22044842

[B18] Stel VS Smit JH Pluijm SM Visser M Deeg DJ Lips P Comparison of the LASA physical acitivity questionnaire with a 7-day diary and pedometer J Clin Epidemiol 2004 57 252 258 10.1016/j.jclinepi.2003.07.008 15066685

[B19] Rosemann T Grol R Herman K Wensing M Szecsenyi J Associtation between obesity, quality of life, physical activity and health service utilization in primary care patients with osteoarthritis Int J Behav Nutr Phys Act 2008 5 4 10.1186/1479-5868-5-4 18226211 PMC2265745

[B20] Kottek M Grieser J Beck C Rudolf B Rubel F World Map of the Köppen-Geiger climate classification updated Meteorol Z 2006 15 259 263 10.1127/0941-2948/2006/0130

[B21] Peel MC Finlayson BL McMahon TA Updated world map of the Köppen-Geiger climate classification Hydrol Earth Syst Sc 2007 11 1633 1644 10.5194/hess-11-1633-2007

[B22] Rolland JS Chronic illness and the life cycle: a conceptual framework Fam Proc 1987 26 203 221 10.1111/j.1545-5300.1987.00203.x 3595826

[B23] Keefe FJ Caldwell DS Queen KT Gil KM Martinez S Crisson JE Ogden W Nunley J Pain coping strategies in osteoarthritis patients J Consult Clin Psychol 1987 55 208 212 3571674 10.1037//0022-006x.55.2.208

[B24] Lasch KE Culture, pain and culturally sensitive pain care Pain Manag Nurs 2000 1 16 22 10.1053/jpmn.2000.9761 11710145

[B25] Rahin-Williams B Riley JL Williams AKK Fillingim RB A quantitative review of ethnic group differences in experimental pain response: do biology, psychology and culture matter? Pain Med 2012 13 522 540 10.1111/j.1526-4637.2012.01336.x 22390201 PMC3349436

